# Molecular identification of *Fasciola* spp. (Digenea: Fasciolidae) in Egypt

**DOI:** 10.1051/parasite/2012192177

**Published:** 2012-05-15

**Authors:** Y. Dar, S. Amer, A. Mercier, B. Courtioux, G. Dreyfuss

**Affiliations:** 1 Zoology Department, Faculty of Science, Tanta University Egypt; 2 Department of Zoology, Faculty of Science, Kafr El-Sheikh University Kafr El Sheikh 33516 Egypt; 3 EA 3174 Tropical and Comparative Neuroepidemiology, Institute of Tropical Neurology, and IFR 145, GEIST, Faculties of Medicine and Pharmacy, University of Limoges France

**Keywords:** *Fasciola gigantica*, *Fasciola hepatica*, Egypt, PCR-RFLP, *Fasciola gigantica*, *Fasciola hepatica*, Égypte, PCR-RFLP

## Abstract

A total of 134 Egyptian liver flukes were collected from different definitive hosts (cattle, sheep, and buffaloes) to identify them via the use of PCR-RFLP and sequence analysis of the first nuclear ribosomal internal transcribed spacer (ITS1). Specimens of *F. hepatica* from France, as well as *F. gigantica* from Cameroon were included in the study for comparison. PCR products of ITS1 were subjected for digestion by *Rsa*I restriction enzyme and visualized on agarose gel. According to RFLP pattern, Egyptian flukes were allocated into two categories. The first was identical to that of French *hepatica* flukes to have a pattern of 360, 100, and 60 (bp) band size, whereas the second resembled to that of Cameroonian *gigantica* worms to have a profile of 360, 170, and 60 bp in size. Results of RFLP analysis were confirmed by sequence analysis of representative ITS1 amplicons. No hybrid forms were detected in the present study. Taken together, this study concluded that both species of *Fasciola* are present in Egypt, whereas the hybrid form may be not very common.

## Abstract (fr)

Des douves égyptiennes (134 spécimens) provenant de différents hôtes définitifs (bovins, buffles et moutons) ont été analysées par PCR-RFLP et l’étude de la séquence ITS-1 pour identifier les espèces locales de *Fasciola* (*F. hepatica*, *F. gigantica*, ou les formes hybrides entre ces deux espèces). Des douves provenant de France (*F. hepatica*) et du Cameroun (*F. gigantica*) ont été utilisées comme références. Deux types de bandes ont été trouvés dans les fragments de la séquence ITS-1 : le premier type est identique à celui des *F. hepatica* de France (trois bandes de 60, 100 et 360 bp) tandis que le second ressemble à celui des *F. gigantica* du Cameroun (trois bandes de 60, 170 et 360 bp). De plus, le séquençage des amplicons ITS-1 confirme cette différence en montrant la présence de six sites nucléotidiques variables, ce qui permet de discriminer *F. hepatica* de *F. gigantica*. Aucune forme intermédiaire entre les deux *Fasciola* n’a été trouvée dans les spécimens analysés. Pris ensemble, cette étude permet de conclure que les deux espèces de *Fasciola* sont présentes en Égypte, alors que la forme hybride pourrait être pas très commune.

The species of genus *Fasciola* (Platyhelmintha: Digenea) are the common liver flukes of a wide range of animals (Mas-Coma *et al.*, 2005). Severe negative economic impact due to fascioliasis has been estimated in ruminants (Urquhart *et al.*, 1996; Spithill & Dalton, 1998). In addition, human fascioliasis represents a significant health problem, as the estimated number of infected people is between 2.4 and 17 million and the number of those at the risk is more than 180 million throughout the world (World Health Organization, 1995; Mas-Coma *et al.*, 1999; Ishii *et al.*, 2002; Mas-Coma *et al.*, 2009). Although, liver flukes have a global geographical distribution, the disease probably exerts most of its impact on developing countries. In Africa, *Fasciola* infection has been recognized as a major constraint for public health and animal farm industry (Mekroud *et al.*, 2006; Pfukenyi *et al.*, 2006; Phiri *et al.*, 2007).

In Egypt, fascioliasis has serious veterinary and medical impacts, as it provokes the mortality of livestock and affects people at all ages (Haseeb *et al.*, 2002; Curtale *et al.*, 2003; Esteban *et al.*, 2003). Coexistence of Egyptian *F. hepatica* and *F. gigantica*, the causative agents of fascioliasis, was determined by Lofty *et al.* (2002) using morphometric and isoelectric focusing techniques. In addition, Marcilla *et al.* (2002) used a PCR-RFLP assay, based on 28S ribosomal DNA, to distinguish between both fasciolids collected from different countries including Egypt. Notably, the occurrence of *F. hepatica*/*F. gigantica* intermediate forms was confirmed in Egypt morphologically (Periago *et al.*, 2008) and molecularly based on ribosomal and mitochondrial gene markers (Amer *et al.*, 2011). Latter findings suggested a possible hybridization between these two species of *Fasciola*, as demonstrated in Japanese, Korean, Chinese and Vietnamese flukes (Itagaki & Tsutsumi, 1998; Agatsuma *et al.*, 2000; Lin *et al.*, 2007; Itagaki *et al.*, 2009). Such hybridization of both fluke species may result in messing up chromosomal numbers with the appearance of diploid and triploid forms with spermic and aspermic (with no or a few spermatozoa in the seminal vesicle) features.

The present study aimed to spot light on speciation of *Fasciola* population in Egypt. *Fasciola* spp. collected from different hosts and localities in Egypt were identified by PCR-RFLP based on ITS1 fragment using *Rsa*I restriction endonuclease. Results of RFLP analysis were confirmed by sequence analysis of representative ITS1 amplicons.

## Materials and Methods

A total of 134 adult *Fasciola* spp. (Table I) were collected from livers of naturally infected buffaloes, cattle and sheep at Al Basateen (Cairo, Egypt), and Tanta (about 90 Km north of Cairo, Egypt) abattoirs during 2008. *F. hepatica* specimens, collected from cattle at the slaughterhouse of Limoges (France), and *F. gigantica*, obtained from cattle at Yaoundé (Cameroon), were included for comparison. Fresh worms were washed extensively in physiological saline and fixed in 70° ethanol.

**Table I.** Total number of analyzed liver flukes from different definitive hosts.

		Distribution of collected flukes			
Species	Origin	Cattle	Sheep	Buffaloes	Total number
*Fasciola hepatica*	Cairo	31	15	–	46
	Tanta	32	15	–	47
	Limoges	3	–	–	3
*Fasciola gigantica*	Cairo	14	15	12	41
	Yaoundé	10	–	–	10
Total number		90	45	12	147

### DNA Extraction and PCR-RFLP Analysis

Genomic DNA was extracted from the adult flukes using QIAamp DNA Mini Kits (Qiagen, USA) following manufacturer’s recommendations. The ITS1 fragment was amplified by PCR (Itagaki *et al.*, 2005), using a set of 5’-TTGCGCTGATTACGTCCCTG-3’ and 5’-TTGGCTGCGCTCTTCATCGAC- 3’ as forward and reverse primers, respectively. The reaction was done in a total volume of 25 μl containing of 12.5 μl Qiagen Multiplex, 1 μl of each primer (0.3 μM), 1 μl genomic DNA, and 9.5 μl H2O. Reaction cycles consisted of an initial denaturation step at 94 °C for 5 min, followed by 30 cycles of denaturation at 94 °C for 90 sec, annealing at 53 °C for 30 sec and extension at 72 °C for 60 sec, followed by a final extension step at 72 °C for 10 min. RFLP reaction was performed using *Rsa*I restriction enzyme as described by Ichikawa and Itagaki (2010). The digested fragments were electrophoresed on 2% agarose gel and visualized by ethidium bromide using GELSMART 7.0 software (Clara Vision). The size of each band was determined by a 100-bp plus ladder molecular weight marker.

### ITS1 Sequencing

For confirmation of RFLP reaction, ITS1 amplicons of representative samples of Egyptian as well as French and Cameroonian flukes were subjected to sequence analysis. PCR products of ITS1 were purified using rapid PCR purification systems (Marligen Bioscience, Inc., USA) according to manufacturer’s instructions. The sequencing reaction was performed utilizing the same PCR primers using ABI Big Dye kit on 3130xl genetic analyzer (Applied Biosystems, France). Accuracy of data was confirmed by two-directional sequencing. Representative sequences were deposited in the GenBank under the accession numbers of JF294998, JF294999, JF295000, and JF295001.

## Results

**Fig. 1.**
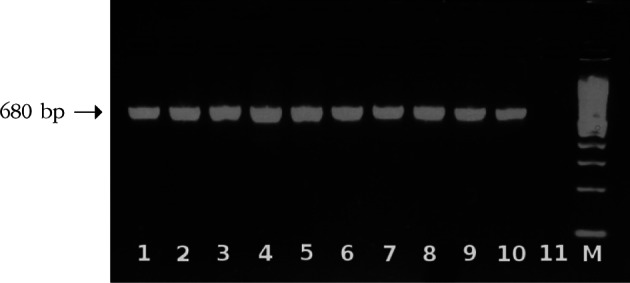
Agarose gel electrophoresis of amplified ITS1 ribosomal region. Lanes 1-10 denote to different fluke samples amplified as a single band of 680 bp; lane 11, negative control; lane M, 100 bp ladder molecular weight marker.

**Fig. 2.**
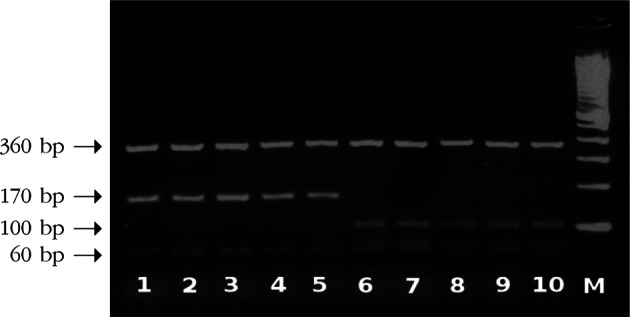
*Rsa*I restriction enzyme digestion products of ITS1 fragment. Lanes 1-5 denote to those of *Fasciola gigantica* isolated from cattle at Yaoundé, Cameron (1) and Cairo, Egypt (2, 3); as well as from sheep at Cairo, Egypt (4, 5), respectively. Lanes 6-10 denote to those of *Fasciola hepatica* isolated from sheep and cattle (6, 7) at Cairo, Egypt, and sheep and cattle (8, 9) at Tanta, Egypt, respectively, as well as from cattle at Limoges, France (10).

PCR amplification of ITS1 resulted in a fragment size of 680 bp (Fig. 1). The products were subsequently subjected to digestion by *Rsa*I restriction enzyme. Electrophoresis of the digested products revealed to two different bands patterns (Fig. 2), regardless of their geographical origins. The first pattern composed of three bands of 360, 100, and 60 bp in size, whereas the second was 360, 170, and 60 bp in size.

**Fig. 3.**
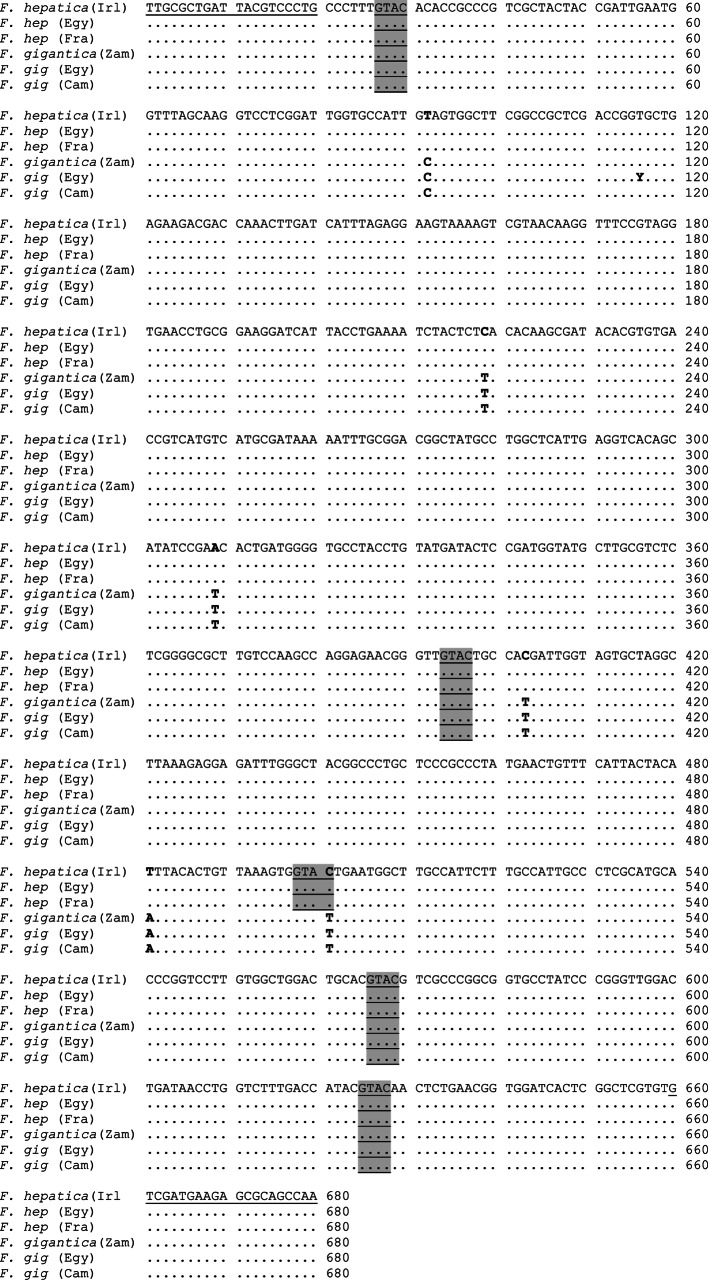
Nucleotide sequence alignment of ITS1 fragments of *Fasciola* species from Egypt (Egy) and four other countries: Cameroon (Cam), France (Fra), Ireland (Irl), and Zambia (Zam). The restriction sites of *Rsa*I endonuclease are underlined and shaded in gray. The nucleotide differences between the two *Fasciola* species are denoted in bold. Dots indicate that the nucleotides are identical to those in the upper line. ‘Y’ represents a nucleotide indicating the presence of a double peak, C and T. (Irl), GenBank accession number: AB514850 for *Fasciola hepatica* from Ireland. (Zam), GenBank accession number: AB514855 for *Fasciola gigantica* from Zambia. Underline denote to primer position.

Alignment of obtained sequences revealed that the members of the first pattern including French flukes belonged to *F. hepatica* type, whereas those of the second pattern including Cameroonian flukes was belonging to *F. gigantica* type (Fig. 3). Six variable nucleotide positions of 92, 219, 309, 403, 481, and 501 were discriminating between nucleotide sequence of both patterns of *Fasciola*. In addition, five restriction sites *Rsa*I enzyme (GTAC) were recognized at positions 27, 394, 498, 566 and 625 in case of *F. hepatica* sequences, whereas four restriction sites were detected at positions 27, 394, 566 and 625 in case of *F. gigantica* sequences. Interestingly, a single nucleotide difference between ITS1 sequences of Cameroonian and Egyptian *F. gigantica* was detected at position 116. At this site, the nucleotide was (T) in the former isolate, whereas it showed a combination of both C and T in the latter one. Collectively, Table I illustrates the identified liver flukes in relation to their origins and their definitive hosts. Out of 134 analyzed specimens from Egypt, 93 (69.4%) flukes were identified as *F. hepatica* and 41 (30.6%) as *F. gigantica*. The latter species was only detected in animals slaughtered at Cairo abattoir, while *F. hepatica* was found in cattle and sheep sacrificed in both abattoirs of Cairo and Tanta.

## Discussion

PCR-RFLP based on ITS1 is a reliable tool to differentiate *F. hepatica* from *F. gigantica* (Ichikawa & Itagaki, 2010; Rokni *et al.*, 2010). In the present study, results showed the presence of two different ITS1-RFLP patterns corresponding to *F. hepatica* (360, 100, and 60 bp band size) and *F. gigantica* (360, 170, and 60 bp band size) as confirmed by PCR-sequence analysis. These results come in full agreement with those described by Ichikawa & Itagaki (2010) for *Fasciola* from several geographical regions. Although nucleotide composition reveals five (*hepatica*) and four (*gigantica*) restriction sites in the affiliated sequences, only three bands in each case were recognized. The band of 60 bp was thought to include the three fragments of 68 bp (only *F. hepatica*), 59 and 54 bp, and the small fragment of 28 bp was not detected on agarose gels (Ichikawa & Itagaki, 2010). Although the intermediate form of *Fasciola* sp. has been reported in Egypt (Amer *et al.*, 2011), the present study did not detect the discriminating pattern (360, 170, 100, and 60 bp band size as described by Ichikawa and Itagaki, 2010) by RFLP reaction nor by sequence analysis. This may indicate that the hybrid form of *Fasciola* is not very common in Egypt and could be detected on sporadic bases. Of note, several PCR-RFLP techniques were developed to differentiate between *F. hepatica* and *F. gigantica* as described by El-Gozamy and Shoukry (2009) using 18S rRNA, Marcilla *et al.* (2002) using 28S rRNA and Rokni *et al.* (2010) using ITS1 utilizing different array of endonucleases. Although nucleotide sequence analysis was consistent with the results of RFLP reaction, Egyptian *F. gigantica* proved heterogeneity at position 116 compared to Cameroonian *gigantica* flukes. Such heterogeneity was reported in Egyptian flukes (Amer *et al.*, 2011). Moreover, nucleotide variability was reported in *Fasciola* flukes (Vara-Del Río *et al.*, 2007, Walker *et al.*, 2011) based on sequence of different genetic markers.

The percentage of *F. hepatica* (69.4%) noted among the slaughtered animals in the present study gave an idea about the adaptation of this fluke to the local environment in Egypt. Occurrence of this digenean, which may have be introduced into this country from Europe through imported infected animals (Lofty *et al.*, 2002; Mas-Coma *et al.*, 2005), was supported by a recent report suggesting the role of *Radix natalensis* (the principal snail host of *F. gigantica*) as a potential intermediate host for *F. hepatica* (Dar *et al.*, 2010). The other fasciolid, *F. gigantica* present in 30.7% in the analyzed samples, was considered as an indigenous species of *Fasciola* found in Egypt (Farag, 1998), as it was recorded in nearly all governorates, especially those of the Nile Delta in Lower Egypt (Lotfy & Hillyer, 2003; Dar *et al.*, 2005).
